# Poly[di­aqua­[μ_2_-1,4-bis­(pyridin-3-ylmeth­yl)piperazine][μ_2_-4-(2-carboxyl­atoeth­yl)benzoato]cobalt(II)]

**DOI:** 10.1107/S2414314624012252

**Published:** 2024-12-24

**Authors:** Frederick C. Ezenyilimba, Robert L. LaDuca

**Affiliations:** a E-35 Holmes Hall, Michigan State University, Lyman Briggs College, 919 E. Shaw Lane, East Lansing, MI 48825, USA; University of Kentucky, USA

**Keywords:** crystal structure, layered coordination polymer, cobalt

## Abstract

A layered cobalt coordination polymer containing both 4-(2-carboxyl­atoeth­yl)benzoate (ceb) and 1,4-bis­(3-pyridyl­meth­yl)piperazine (3-bpmp) ligands, [Co(C_10_H_8_O_4_)(C_16_H_20_N_4_)(H_2_O)_2_]_*n*_ or [Co(ceb)(3-bpmp)(H_2_O)_2_]_*n*_, was isolated and structurally characterized by single-crystal X-ray diffraction. Chain-like [Co(ceb)(H_2_O)_2_]_*n*_ units are oriented parallel to [101]. These are connected into (4,4)-grid coordination polymer layers by tethering 3-bpmp ligands. The layer motifs stack in an *AAA* pattern mediated by O—H⋯N hydrogen-bonding inter­actions between the aqua ligands in one layer and 3-bpmp piperazinyl N atoms in the abutting layer.

## Abstract 2

A layered cobalt coordination polymer containing both 4-(2-carboxyl­atoeth­yl)benzoate (ceb) and *N*,*N*′-bis­(3-pyridyl­meth­yl)piperazine (3-bpmp) ligands, [Co(ceb)(3-bpmp)(H_2_O)_2_]_*n*_, was isolated and structurally characterized by single-crystal X-ray diffraction.

## Graphical abstract

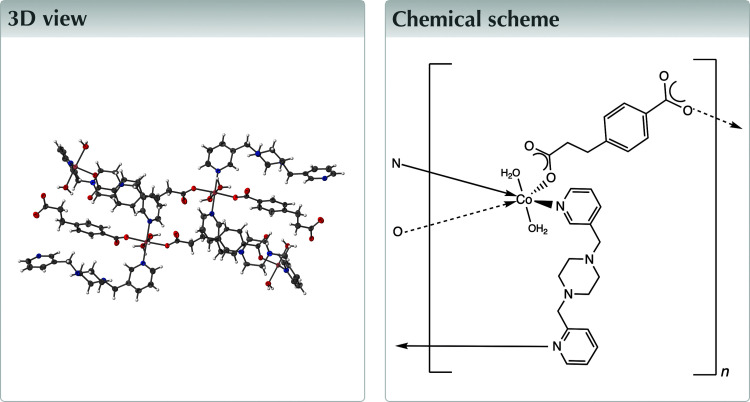

## Structure description

The title compound was isolated during an exploratory synthetic effort aiming to produce a cobalt coordination polymer containing both 4-(2-carboxyl­atoeth­yl)benzoate (ceb) and *N*,*N*′-bis­(3-pyridyl­meth­yl)piperazine (3-bpmp) ligands. Zinc pyromellitate coordination polymers containing the 3-bpmp ligand and its related congener *N*,*N*′-bis­(4-pyridyl­meth­yl)piperazine (4-bpmp) exhibited intriguing and diverse self-penetrated topologies (Blake *et al.*, 2011 ▸).

**Figure 1:**
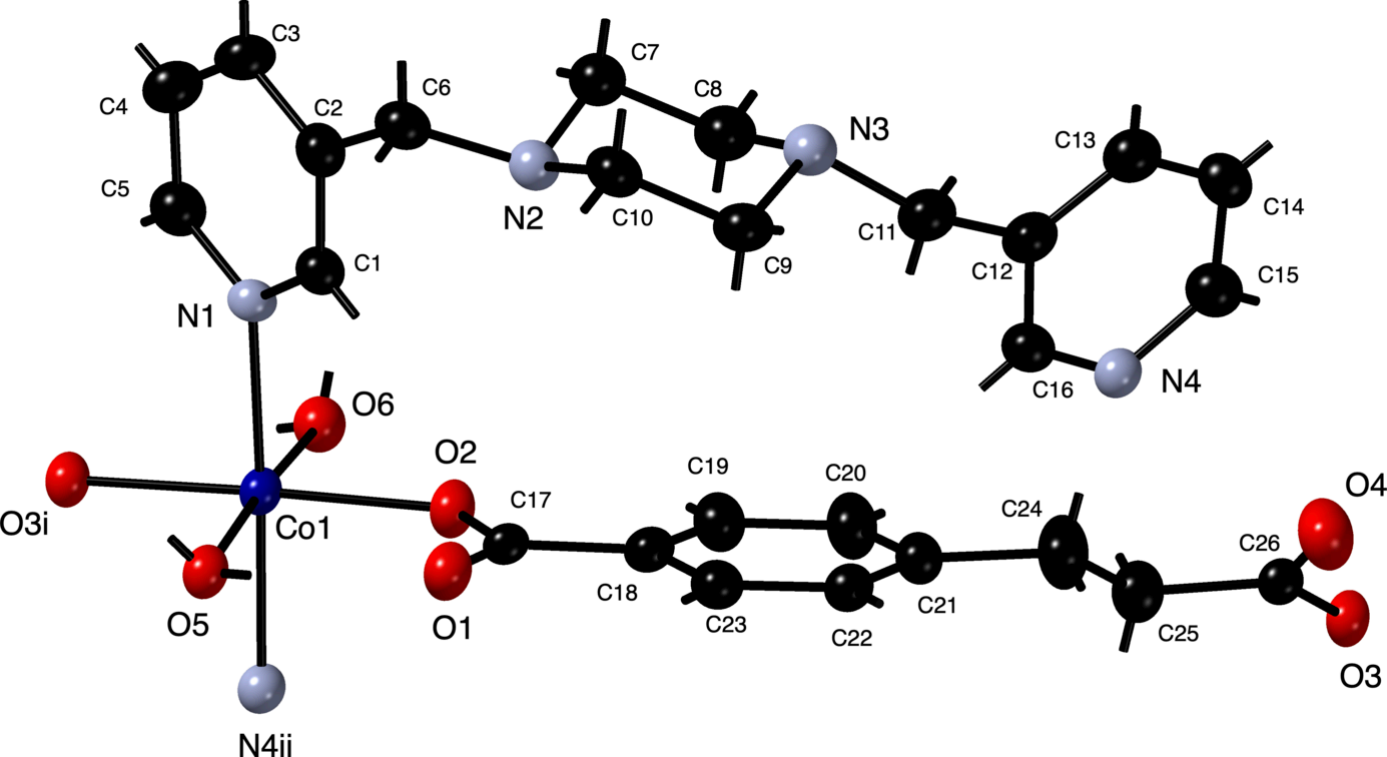
The coordination environment of the title compound, showing octa­hedral coordination at the Co1 atom. Displacement ellipsoids are drawn at the 50% probability level. Color code: Co, dark blue, N, light blue; O, red; C, black. H atom positions are shown as sticks.

The asymmetric unit of the title compound consists of a divalent Co atom, a fully deprotonated ceb ligand, a 3-bpmp ligand, and two bound water mol­ecules. The Co atom displays a {CoO_4_N_2_} octa­hedral coordination environment (Fig. 1 ▸) with two *trans* pyridyl N-atom donors belonging to two 3-bpmp ligands, and two *trans* aqua ligands. The two remaining *trans* coordination sites are occupied by carboxyl­ate O atoms belonging to two ceb ligands, one from a shorter carboxyl­ate terminus, and one from the longer three-C-atom carboxyl­ate arm. Bond lengths and angles within the coordination environment are consistent with octa­hedral coordination without any chelating ligands at the Co atoms (Table 1 ▸).

**Figure 2:**
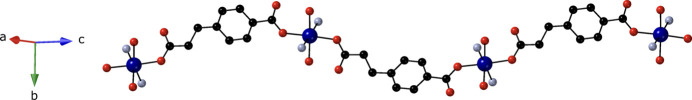
[Co(ceb)(H_2_O)_2_]_*n*_ coordination polymer chain in the title compound, oriented parallel to [10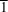].

**Figure 3:**
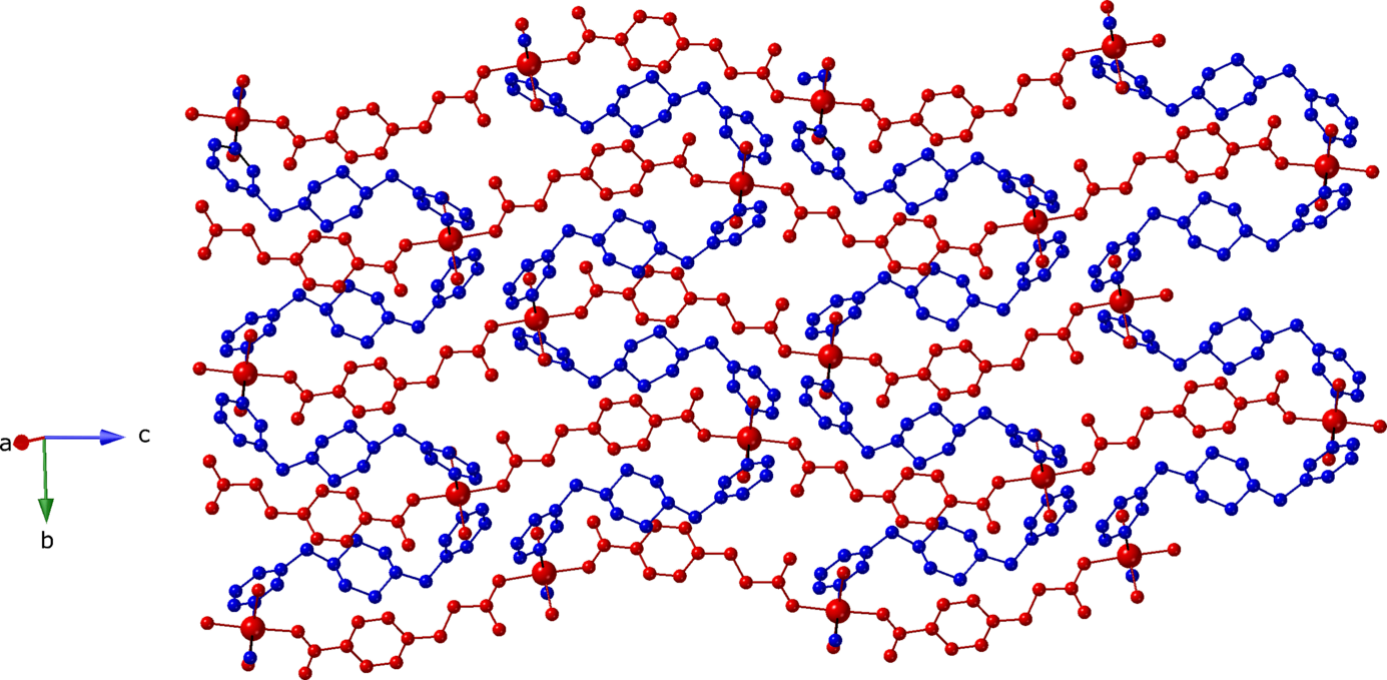
[Co(ceb)(3-bpmp)(H_2_O)_2_)]_*n*_ coordination polymer layer in the title compound. [Co(ceb)(H_2_O)_2_]_*n*_ coordination polymer chains are drawn in red, and the 3-bpmp linkers are drawn in blue.

**Figure 4:**
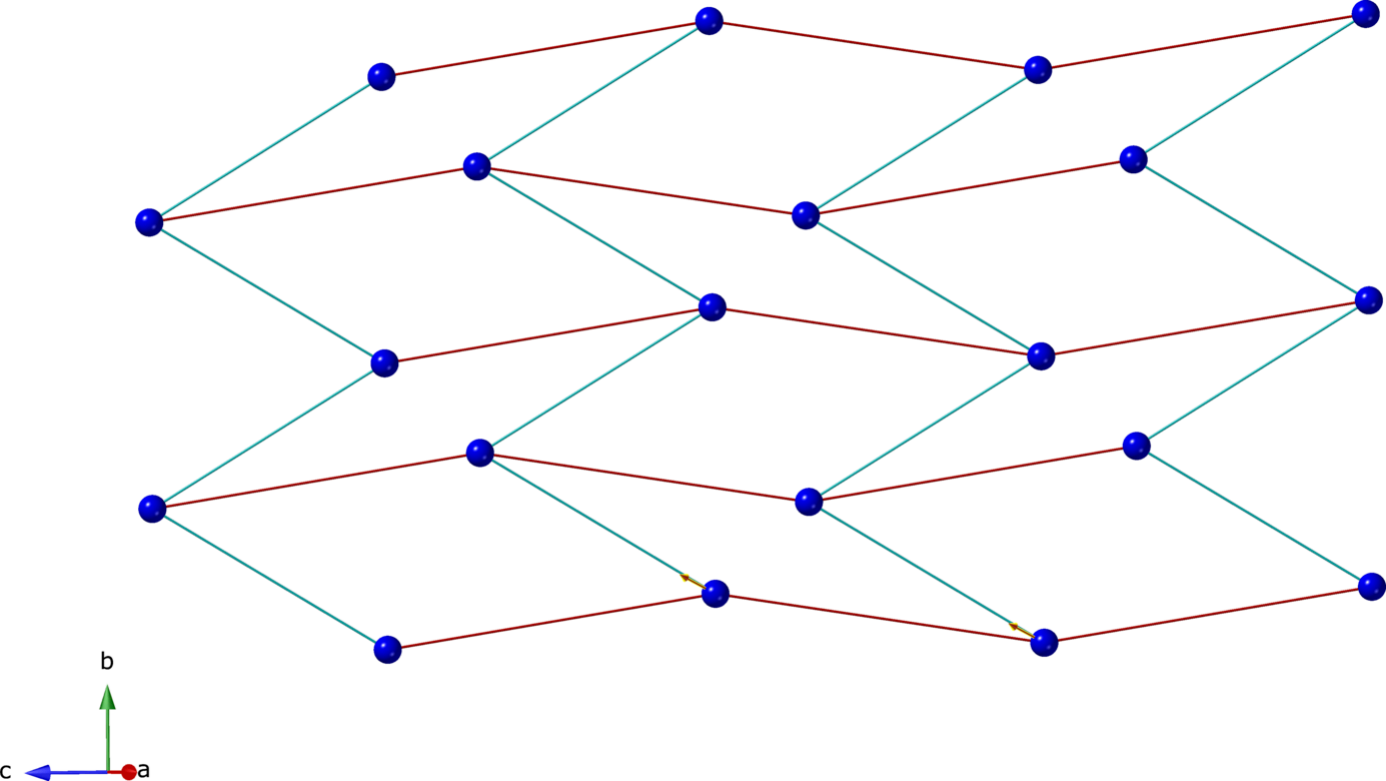
Schematic representation of the (4,4) grid layer motif in the title compound. The dark blue spheres represent the Co^II^ ions. Red rods represent the ceb ligands, and blue rods represent the 3-bpmp linkers.

**Figure 5:**
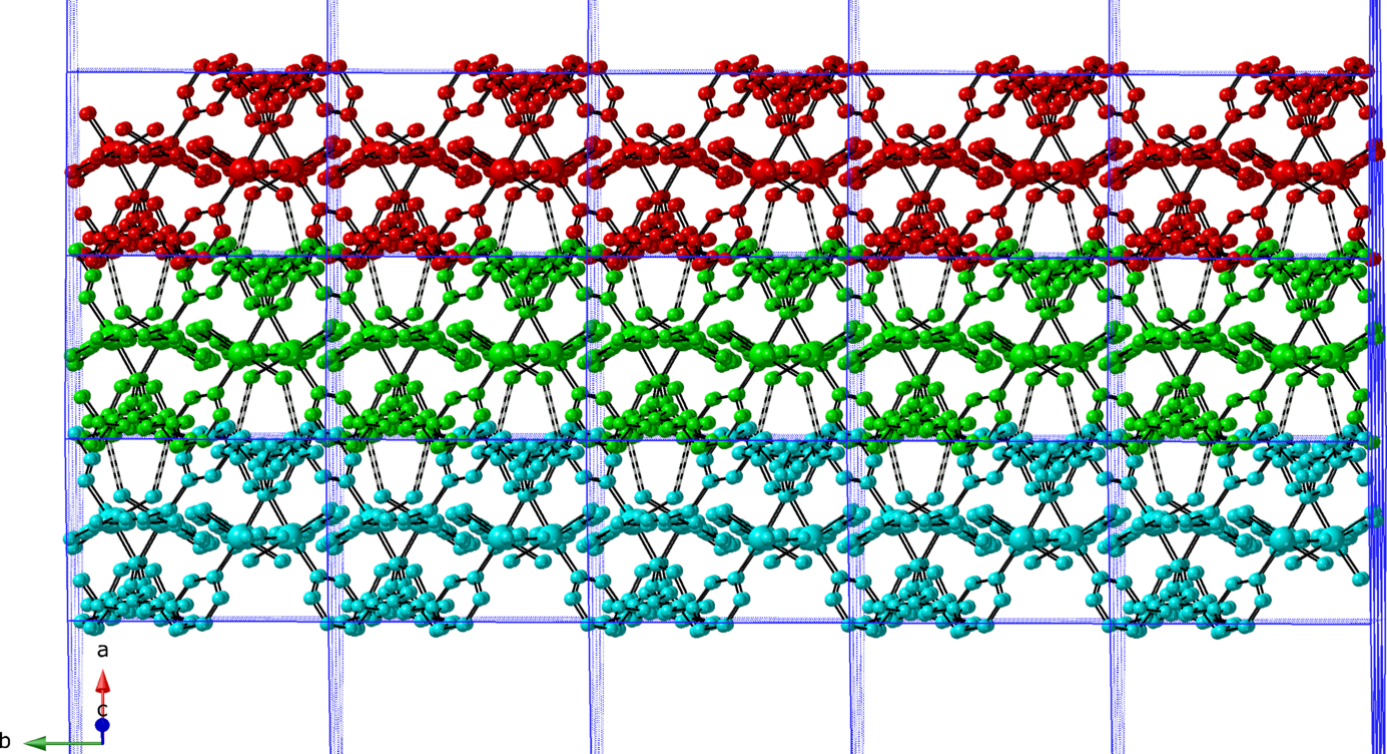
*AAA* parallel stacking of supra­molecular layer motifs in the title compound, mediated by inter­layer O—H⋯N hydrogen-bonding inter­actions, which are shown as dashed lines.

Adjacent Co atoms are linked by bis­(monodentate) ceb ligands, thereby constructing mono-periodic [Co(ceb)(H_2_O)_2_]_*n*_ coordination polymer chains (Fig. 2 ▸), which are oriented parallel to [10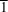]. Intra-chain O—H⋯O hydrogen bonding is observed between the aqua ligands and unligated ceb carboxyl­ate O atoms (Table 2 ▸). The chain motifs are linked into [Co(ceb)(3-bpmp)(H_2_O)_2_)]_*n*_ coordination polymer layers by tethering 3-bpmp ligands (Fig. 3 ▸). Treating the Co atoms as four-connected nodes with ceb and 3-bpmp rod-like linkers reveals a (4,4) grid network with parallelogram apertures (Fig. 4 ▸). Adjacent [Co(ceb)(3-bpmp)(H_2_O)_2_)]_*n*_ coordination polymer layers form the complete three-dimensional crystal structure of the title compound by means of *AAA* parallel stacking along the *a*-axis direction. The stacking is mediated by inter­layer O—H⋯N hydrogen-bonding inter­actions between the aqua ligands in one layer and 3-bpmp piperazinyl N atoms in the adjacent layer (Fig. 5 ▸, Table 2 ▸).

## Synthesis and crystallization

Co(NO_3_)_2_·6H_2_O (108 mg, 0.37 mmol), 4-(2-carboxyl­atoeth­yl)benzoic acid (72 mg, 0.37 mmol), 3-bpmp (110 mg, 0.37 mmol) and 0.75 ml of a 1.0 *M* NaOH solution were placed into 10 ml of distilled H_2_O in a Teflon-lined acid digestion bomb. The bomb was sealed and heated in an oven at 393 K for 2 d, and then cooled slowly to 273 K. Pale-orange crystals of the title complex were isolated after washing with distilled water and acetone, and drying in air.

## Refinement

Crystal data, data collection and structure refinement details are summarized in Table 3 ▸.

## Supplementary Material

Crystal structure: contains datablock(s) I. DOI: 10.1107/S2414314624012252/pk4046sup1.cif

Structure factors: contains datablock(s) I. DOI: 10.1107/S2414314624012252/pk4046Isup3.hkl

CCDC reference: 1977424

Additional supporting information:  crystallographic information; 3D view; checkCIF report

## Figures and Tables

**Table 1:** Selected geometric parameters (Å, °)

Co1—O1	2.068 (3)	Co1—O6	2.135 (3)
Co1—O3^i^	2.099 (3)	Co1—N1	2.172 (4)
Co1—O5	2.138 (3)	Co1—N4^ii^	2.176 (4)
			
O1—Co1—O3^i^	177.25 (12)	O3^i^—Co1—N4^ii^	88.62 (13)
O1—Co1—O5	87.36 (12)	O5—Co1—N1	93.73 (14)
O1—Co1—O6	91.60 (12)	O5—Co1—N4^ii^	91.36 (14)
O1—Co1—N1	91.83 (14)	O6—Co1—O5	175.84 (12)
O1—Co1—N4^ii^	89.08 (14)	O6—Co1—N1	90.33 (14)
O3^i^—Co1—O5	91.19 (12)	O6—Co1—N4^ii^	84.59 (13)
O3^i^—Co1—O6	89.69 (12)	N1—Co1—N4^ii^	174.87 (14)
O3^i^—Co1—N1	90.59 (13)		

Symmetry codes: (i) 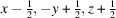; (ii) 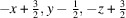.

**Table 2:** Hydrogen-bond geometry (Å, °)

*D*—H⋯*A*	*D*—H	H⋯*A*	*D*⋯*A*	*D*—H⋯*A*
O5—H5*A*⋯N3^iii^	0.89	2.20	2.980 (5)	146
O5—H5*B*⋯O4^i^	0.89	1.81	2.600 (5)	147
O6—H6*A*⋯O3^iv^	0.90	1.92	2.760 (4)	154
O6—H6*B*⋯O2	0.90	1.84	2.641 (4)	147

Symmetry codes: (i) 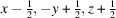; (iii) 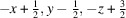; (iv) 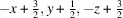.

**Table 3:** Experimental details

Crystal data	
Chemical formula	[Co(C_10_H_8_O_4_)(C_16_H_20_N_4_)(H_2_O)_2_]
*M*_r_	555.48
Crystal system, space group	Monoclinic, *P*2_1_/*n*
Temperature (K)	173
*a*, *b*, *c* (Å)	8.792 (5), 11.674 (7), 24.976 (14)
β (°)	92.06 (2)
*V* (Å^3^)	2562 (2)
*Z*	4
Radiation type	Mo *K*α
μ (mm^−1^)	0.72
Crystal size (mm)	0.15 × 0.14 × 0.10

Data collection	
Diffractometer	Bruker APEXII CCD
Absorption correction	Multi-scan (*SADABS*; Krause *et al.*, 2015 ▸)
*T*_min_, *T*_max_	0.619, 0.745
No. of measured, independent and observed [*I* > 2σ(*I*)] reflections	18006, 4662, 2979
*R*_int_	0.101
(sin θ/λ)_max_ (Å^−1^)	0.602

Refinement	
*R*[*F*^2^ > 2σ(*F*^2^)], *wR*(*F*^2^), *S*	0.064, 0.143, 1.02
No. of reflections	4662
No. of parameters	336
H-atom treatment	H-atom parameters constrained
Δρ_max_, Δρ_min_ (e Å^−3^)	0.82, −0.32

Computer programs: *COSMO* (Bruker, 2009 ▸), *SAINT* (Bruker, 2014 ▸), *SHELXT* (Sheldrick, 2015*a* ▸), *SHELXL* (Sheldrick, 2015*b* ▸), *OLEX2* (Dolomanov *et al.*, 2009 ▸) and *CrystalMaker X* (Palmer, 2020 ▸).

## full crystallographic data

### Poly[diaqua[µ2-1,4-bis(pyridin-3-ylmethyl)piperazine][µ2-4-(2-carboxylatoethyl)benzoato]cobalt(II)] Crystal data

**Table:**

[Co(C_10_H_8_O_4_)(C_16_H_20_N_4_)(H_2_O)_2_]	*F*(000) = 1164
*M**_r_* = 555.48	*D*_x_ = 1.440 Mg m^−^^3^
Monoclinic, *P*2_1_/*n*	Mo *K*α radiation, λ = 0.71073 Å
*a* = 8.792 (5) Å	Cell parameters from 3606 reflections
*b* = 11.674 (7) Å	θ = 2.4–25.2°
*c* = 24.976 (14) Å	µ = 0.72 mm^−^^1^
β = 92.06 (2)°	*T* = 173 K
*V* = 2562 (2) Å^3^	Block, orange
*Z* = 4	0.15 × 0.14 × 0.10 mm

### Poly[diaqua[µ2-1,4-bis(pyridin-3-ylmethyl)piperazine][µ2-4-(2-carboxylatoethyl)benzoato]cobalt(II)] Data collection

**Table:**

Bruker APEXII CCD diffractometer	4662 independent reflections
Radiation source: sealed tube	2979 reflections with *I* > 2σ(*I*)
Graphite monochromator	*R*_int_ = 0.101
Detector resolution: 8.36 pixels mm^-1^	θ_max_ = 25.3°, θ_min_ = 1.6°
ω scans	*h* = −10→10
Absorption correction: multi-scan (SADABS; Krause *et al.*, 2015)	*k* = −14→14
*T*_min_ = 0.619, *T*_max_ = 0.745	*l* = −29→29
18006 measured reflections	

### Poly[diaqua[µ2-1,4-bis(pyridin-3-ylmethyl)piperazine][µ2-4-(2-carboxylatoethyl)benzoato]cobalt(II)] Refinement

**Table:**

Refinement on *F*^2^	Primary atom site location: dual
Least-squares matrix: full	Hydrogen site location: mixed
*R*[*F*^2^ > 2σ(*F*^2^)] = 0.064	H-atom parameters constrained
*wR*(*F*^2^) = 0.143	*w* = 1/[σ^2^(*F*_o_^2^) + (0.0562*P*)^2^ + 1.877*P*] where *P* = (*F*_o_^2^ + 2*F*_c_^2^)/3
*S* = 1.02	(Δ/σ)_max_ = 0.001
4662 reflections	Δρ_max_ = 0.82 e Å^−^^3^
336 parameters	Δρ_min_ = −0.32 e Å^−^^3^
0 restraints	

### Poly[diaqua[µ2-1,4-bis(pyridin-3-ylmethyl)piperazine][µ2-4-(2-carboxylatoethyl)benzoato]cobalt(II)] Special details

**Table:**

Experimental. Data was collected using a Bruker CCD (charge coupled device) based diffractometer equipped with an Oxford low-temperature apparatus operating at 173 K. A suitable crystal was chosen and mounted on a nylon loop using Paratone oil. Data were measured using omega scans of 0.5° per frame for 30 s. The total number of images were based on results from the program *COSMO* (Bruker, 2009) where redundancy was expected to be 4 and completeness to 0.83Å to 100%. Cell parameters were retrieved using APEX II software and refined using *SAINT* (Bruker, 2014) on all observed reflections. Data reduction was performed using the *SAINT* software, which corrects for Lorentz/polarization effects. Scaling and absorption corrections were applied using *SADABS* (Krause *et al.*, 2015). The structure was solved by the dual-space direct methods program *SHELXT* program and refined by least squares method on F^2^ using *SHELXL*, called from within *OLEX2*.
Geometry. All esds (except the esd in the dihedral angle between two l.s. planes) are estimated using the full covariance matrix. The cell esds are taken into account individually in the estimation of esds in distances, angles and torsion angles; correlations between esds in cell parameters are only used when they are defined by crystal symmetry. An approximate (isotropic) treatment of cell esds is used for estimating esds involving l.s. planes.
Refinement. The structure was refined by Least Squares using version 2018/3 of *SHELXL* (Sheldrick, 2015) incorporated in *Olex2* (Dolomanov *et al.*, 2009). All non-hydrogen atoms were refined anisotropically. Hydrogen atom positions were calculated geometrically and refined using the riding model, except for the hydrogen atom on the nitrogen atom which was found by difference Fourier methods and refined isotropically.

### Poly[diaqua[µ2-1,4-bis(pyridin-3-ylmethyl)piperazine][µ2-4-(2-carboxylatoethyl)benzoato]cobalt(II)] Fractional atomic coordinates and isotropic or equivalent isotropic displacement parameters (Å^2^)

**Table:**

	*x*	*y*	*z*	*U*_iso_*/*U*_eq_	
Co1	0.53449 (7)	0.34158 (5)	0.92351 (2)	0.02444 (19)	
O1	0.6159 (4)	0.3598 (3)	0.84732 (12)	0.0299 (8)	
O2	0.7352 (4)	0.5298 (3)	0.85176 (12)	0.0318 (8)	
O3	0.9573 (3)	0.1845 (2)	0.50111 (12)	0.0284 (8)	
O4	0.9032 (4)	0.3718 (3)	0.49603 (14)	0.0423 (9)	
O5	0.4295 (4)	0.1859 (2)	0.89624 (13)	0.0315 (8)	
H5A	0.338740	0.199708	0.880412	0.047*	
H5B	0.406879	0.142008	0.924053	0.047*	
O6	0.6547 (3)	0.4905 (2)	0.95095 (12)	0.0291 (8)	
H6A	0.591789	0.541260	0.965697	0.044*	
H6B	0.691265	0.529733	0.923306	0.044*	
N1	0.3355 (4)	0.4453 (3)	0.90337 (15)	0.0261 (9)	
N2	0.3174 (4)	0.6616 (3)	0.76873 (15)	0.0297 (9)	
N3	0.3692 (4)	0.6242 (3)	0.65617 (15)	0.0294 (9)	
N4	0.7573 (4)	0.7512 (3)	0.55286 (15)	0.0271 (9)	
C1	0.3447 (5)	0.5353 (4)	0.87023 (18)	0.0288 (11)	
H1	0.441096	0.552813	0.856274	0.035*	
C2	0.2216 (6)	0.6045 (4)	0.85506 (19)	0.0312 (11)	
C3	0.0823 (6)	0.5764 (4)	0.8761 (2)	0.0375 (13)	
H3	−0.005592	0.620111	0.866441	0.045*	
C4	0.0712 (6)	0.4852 (4)	0.9110 (2)	0.0400 (13)	
H4	−0.023554	0.466457	0.925921	0.048*	
C5	0.1990 (6)	0.4222 (4)	0.9238 (2)	0.0352 (12)	
H5	0.191057	0.359847	0.948039	0.042*	
C6	0.2433 (6)	0.7020 (4)	0.81655 (19)	0.0332 (12)	
H6C	0.143248	0.735773	0.806241	0.040*	
H6D	0.306422	0.762289	0.834197	0.040*	
C7	0.2151 (5)	0.5910 (4)	0.73545 (19)	0.0328 (12)	
H7A	0.131568	0.638943	0.720073	0.039*	
H7B	0.169726	0.530620	0.757666	0.039*	
C8	0.3007 (6)	0.5366 (4)	0.6910 (2)	0.0349 (12)	
H8A	0.382057	0.486926	0.706547	0.042*	
H8B	0.230327	0.487942	0.669095	0.042*	
C9	0.4633 (5)	0.7035 (4)	0.6898 (2)	0.0323 (12)	
H9A	0.501062	0.766223	0.667111	0.039*	
H9B	0.552679	0.661682	0.705098	0.039*	
C10	0.3739 (5)	0.7538 (4)	0.73472 (19)	0.0302 (11)	
H10A	0.439769	0.805989	0.756493	0.036*	
H10B	0.286994	0.798696	0.719593	0.036*	
C11	0.4631 (6)	0.5622 (4)	0.6176 (2)	0.0340 (12)	
H11A	0.397652	0.505599	0.598340	0.041*	
H11B	0.542839	0.519076	0.638044	0.041*	
C12	0.5397 (5)	0.6355 (4)	0.57655 (19)	0.0294 (11)	
C13	0.4773 (6)	0.6520 (4)	0.5257 (2)	0.0379 (12)	
H13	0.382732	0.617339	0.515730	0.045*	
C14	0.5520 (6)	0.7189 (4)	0.4891 (2)	0.0399 (13)	
H14	0.508523	0.731996	0.454235	0.048*	
C15	0.6912 (6)	0.7662 (4)	0.5044 (2)	0.0363 (12)	
H15	0.742439	0.811563	0.479093	0.044*	
C16	0.6830 (5)	0.6862 (4)	0.5880 (2)	0.0315 (12)	
H16	0.729698	0.673734	0.622376	0.038*	
C17	0.6953 (5)	0.4403 (4)	0.82744 (18)	0.0264 (11)	
C18	0.7392 (5)	0.4246 (4)	0.77044 (19)	0.0262 (11)	
C19	0.7005 (5)	0.3283 (4)	0.74147 (19)	0.0328 (12)	
H19	0.645341	0.268996	0.758118	0.039*	
C20	0.7400 (6)	0.3154 (4)	0.6885 (2)	0.0380 (13)	
H20	0.711348	0.247664	0.669637	0.046*	
C21	0.8213 (5)	0.4006 (4)	0.66270 (18)	0.0294 (11)	
C22	0.8622 (5)	0.4969 (4)	0.69193 (19)	0.0278 (11)	
H22	0.918031	0.555962	0.675365	0.033*	
C23	0.8237 (5)	0.5091 (4)	0.74467 (19)	0.0281 (11)	
H23	0.854886	0.575828	0.763897	0.034*	
C24	0.8544 (7)	0.3947 (4)	0.6038 (2)	0.0413 (14)	
H24A	0.764799	0.425468	0.583367	0.050*	
H24B	0.940819	0.446660	0.597300	0.050*	
C25	0.8912 (6)	0.2806 (4)	0.5808 (2)	0.0408 (13)	
H25A	0.805773	0.227804	0.587420	0.049*	
H25B	0.982430	0.250048	0.600364	0.049*	
C26	0.9205 (5)	0.2796 (4)	0.52184 (19)	0.0295 (11)	

### Poly[diaqua[µ2-1,4-bis(pyridin-3-ylmethyl)piperazine][µ2-4-(2-carboxylatoethyl)benzoato]cobalt(II)] Atomic displacement parameters (Å^2^)

**Table:**

	*U*^11^	*U*^22^	*U*^33^	*U*^12^	*U*^13^	*U*^23^
Co1	0.0261 (3)	0.0250 (3)	0.0225 (3)	0.0008 (3)	0.0044 (3)	0.0005 (3)
O1	0.0378 (19)	0.0258 (18)	0.0265 (18)	−0.0025 (15)	0.0079 (15)	−0.0003 (14)
O2	0.035 (2)	0.0297 (19)	0.0307 (19)	−0.0084 (15)	0.0075 (16)	−0.0053 (15)
O3	0.0359 (19)	0.0250 (18)	0.0247 (17)	−0.0018 (15)	0.0067 (15)	−0.0028 (14)
O4	0.066 (3)	0.026 (2)	0.035 (2)	0.0050 (17)	0.0016 (19)	−0.0022 (16)
O5	0.0353 (19)	0.0271 (18)	0.0321 (19)	−0.0044 (15)	0.0036 (15)	0.0026 (14)
O6	0.0328 (19)	0.0280 (18)	0.0269 (19)	0.0002 (14)	0.0068 (15)	0.0006 (14)
N1	0.023 (2)	0.029 (2)	0.027 (2)	0.0016 (17)	−0.0019 (17)	0.0027 (17)
N2	0.031 (2)	0.029 (2)	0.029 (2)	−0.0005 (19)	−0.0005 (18)	0.0021 (19)
N3	0.031 (2)	0.023 (2)	0.033 (2)	−0.0023 (17)	0.0009 (19)	0.0033 (17)
N4	0.028 (2)	0.025 (2)	0.028 (2)	0.0009 (18)	0.0041 (19)	−0.0009 (17)
C1	0.030 (3)	0.030 (3)	0.027 (3)	0.003 (2)	−0.001 (2)	0.001 (2)
C2	0.037 (3)	0.028 (3)	0.028 (3)	0.001 (2)	−0.001 (2)	−0.003 (2)
C3	0.026 (3)	0.042 (3)	0.044 (3)	0.005 (2)	−0.001 (2)	0.003 (3)
C4	0.029 (3)	0.048 (3)	0.044 (3)	−0.001 (3)	0.005 (2)	0.006 (3)
C5	0.035 (3)	0.035 (3)	0.035 (3)	−0.006 (2)	−0.001 (2)	0.003 (2)
C6	0.031 (3)	0.034 (3)	0.034 (3)	0.001 (2)	−0.005 (2)	0.001 (2)
C7	0.031 (3)	0.030 (3)	0.037 (3)	−0.007 (2)	0.000 (2)	0.001 (2)
C8	0.033 (3)	0.027 (3)	0.044 (3)	−0.011 (2)	0.000 (2)	0.003 (2)
C9	0.027 (3)	0.029 (3)	0.041 (3)	−0.006 (2)	−0.002 (2)	0.005 (2)
C10	0.032 (3)	0.025 (3)	0.033 (3)	−0.007 (2)	−0.006 (2)	0.003 (2)
C11	0.033 (3)	0.028 (3)	0.041 (3)	0.003 (2)	0.002 (2)	0.001 (2)
C12	0.030 (3)	0.019 (2)	0.039 (3)	0.002 (2)	0.004 (2)	−0.003 (2)
C13	0.030 (3)	0.041 (3)	0.042 (3)	−0.006 (3)	0.002 (2)	−0.006 (3)
C14	0.037 (3)	0.051 (3)	0.031 (3)	−0.003 (3)	−0.004 (3)	0.001 (3)
C15	0.036 (3)	0.036 (3)	0.037 (3)	0.000 (2)	0.000 (3)	0.005 (2)
C16	0.033 (3)	0.030 (3)	0.032 (3)	0.006 (2)	−0.002 (2)	0.003 (2)
C17	0.021 (3)	0.032 (3)	0.026 (3)	0.005 (2)	0.002 (2)	0.001 (2)
C18	0.025 (3)	0.023 (2)	0.031 (3)	0.003 (2)	0.002 (2)	0.000 (2)
C19	0.040 (3)	0.029 (3)	0.030 (3)	−0.007 (2)	0.004 (2)	0.008 (2)
C20	0.050 (3)	0.033 (3)	0.031 (3)	−0.009 (2)	0.004 (3)	−0.007 (2)
C21	0.034 (3)	0.028 (3)	0.026 (3)	0.003 (2)	0.001 (2)	−0.001 (2)
C22	0.025 (3)	0.029 (3)	0.029 (3)	−0.004 (2)	0.004 (2)	0.005 (2)
C23	0.029 (3)	0.023 (3)	0.032 (3)	−0.002 (2)	−0.001 (2)	−0.002 (2)
C24	0.062 (4)	0.030 (3)	0.032 (3)	−0.009 (3)	0.009 (3)	−0.003 (2)
C25	0.048 (3)	0.042 (3)	0.033 (3)	0.008 (3)	0.005 (3)	0.001 (2)
C26	0.023 (3)	0.039 (3)	0.026 (3)	−0.003 (2)	0.002 (2)	−0.005 (2)

### Poly[diaqua[µ2-1,4-bis(pyridin-3-ylmethyl)piperazine][µ2-4-(2-carboxylatoethyl)benzoato]cobalt(II)] Geometric parameters (Å, º)

**Table:**

Co1—O1	2.068 (3)	C7—C8	1.504 (7)
Co1—O3^i^	2.099 (3)	C8—H8A	0.9900
Co1—O5	2.138 (3)	C8—H8B	0.9900
Co1—O6	2.135 (3)	C9—H9A	0.9900
Co1—N1	2.172 (4)	C9—H9B	0.9900
Co1—N4^ii^	2.176 (4)	C9—C10	1.513 (6)
O1—C17	1.282 (5)	C10—H10A	0.9900
O2—C17	1.252 (5)	C10—H10B	0.9900
O3—C26	1.272 (5)	C11—H11A	0.9900
O4—C26	1.261 (6)	C11—H11B	0.9900
O5—H5A	0.8924	C11—C12	1.511 (6)
O5—H5B	0.8915	C12—C13	1.378 (7)
O6—H6A	0.8985	C12—C16	1.412 (7)
O6—H6B	0.8977	C13—H13	0.9500
N1—C1	1.342 (6)	C13—C14	1.386 (7)
N1—C5	1.349 (6)	C14—H14	0.9500
N2—C6	1.459 (6)	C14—C15	1.384 (7)
N2—C7	1.458 (6)	C15—H15	0.9500
N2—C10	1.469 (6)	C16—H16	0.9500
N3—C8	1.484 (6)	C17—C18	1.500 (6)
N3—C9	1.482 (6)	C18—C19	1.373 (6)
N3—C11	1.480 (6)	C18—C23	1.406 (6)
N4—C15	1.336 (6)	C19—H19	0.9500
N4—C16	1.346 (6)	C19—C20	1.387 (6)
C1—H1	0.9500	C20—H20	0.9500
C1—C2	1.393 (6)	C20—C21	1.397 (7)
C2—C3	1.389 (7)	C21—C22	1.381 (6)
C2—C6	1.507 (7)	C21—C24	1.512 (7)
C3—H3	0.9500	C22—H22	0.9500
C3—C4	1.383 (7)	C22—C23	1.379 (6)
C4—H4	0.9500	C23—H23	0.9500
C4—C5	1.371 (7)	C24—H24A	0.9900
C5—H5	0.9500	C24—H24B	0.9900
C6—H6C	0.9900	C24—C25	1.490 (7)
C6—H6D	0.9900	C25—H25A	0.9900
C7—H7A	0.9900	C25—H25B	0.9900
C7—H7B	0.9900	C25—C26	1.505 (7)
			
O1—Co1—O3^i^	177.25 (12)	N3—C9—H9B	109.3
O1—Co1—O5	87.36 (12)	N3—C9—C10	111.6 (4)
O1—Co1—O6	91.60 (12)	H9A—C9—H9B	108.0
O1—Co1—N1	91.83 (14)	C10—C9—H9A	109.3
O1—Co1—N4^ii^	89.08 (14)	C10—C9—H9B	109.3
O3^i^—Co1—O5	91.19 (12)	N2—C10—C9	109.9 (4)
O3^i^—Co1—O6	89.69 (12)	N2—C10—H10A	109.7
O3^i^—Co1—N1	90.59 (13)	N2—C10—H10B	109.7
O3^i^—Co1—N4^ii^	88.62 (13)	C9—C10—H10A	109.7
O5—Co1—N1	93.73 (14)	C9—C10—H10B	109.7
O5—Co1—N4^ii^	91.36 (14)	H10A—C10—H10B	108.2
O6—Co1—O5	175.84 (12)	N3—C11—H11A	108.3
O6—Co1—N1	90.33 (14)	N3—C11—H11B	108.3
O6—Co1—N4^ii^	84.59 (13)	N3—C11—C12	115.9 (4)
N1—Co1—N4^ii^	174.87 (14)	H11A—C11—H11B	107.4
C17—O1—Co1	130.0 (3)	C12—C11—H11A	108.3
C26—O3—Co1^iii^	126.7 (3)	C12—C11—H11B	108.3
Co1—O5—H5A	110.9	C13—C12—C11	122.1 (4)
Co1—O5—H5B	110.3	C13—C12—C16	116.7 (4)
H5A—O5—H5B	103.2	C16—C12—C11	121.1 (4)
Co1—O6—H6A	111.3	C12—C13—H13	119.8
Co1—O6—H6B	110.8	C12—C13—C14	120.3 (5)
H6A—O6—H6B	102.8	C14—C13—H13	119.8
C1—N1—Co1	120.9 (3)	C13—C14—H14	120.7
C1—N1—C5	117.7 (4)	C15—C14—C13	118.5 (5)
C5—N1—Co1	121.4 (3)	C15—C14—H14	120.7
C6—N2—C10	114.0 (4)	N4—C15—C14	123.3 (5)
C7—N2—C6	111.4 (4)	N4—C15—H15	118.3
C7—N2—C10	107.4 (4)	C14—C15—H15	118.3
C9—N3—C8	109.2 (4)	N4—C16—C12	123.7 (4)
C11—N3—C8	107.0 (4)	N4—C16—H16	118.2
C11—N3—C9	111.1 (4)	C12—C16—H16	118.2
C15—N4—Co1^iv^	121.1 (3)	O1—C17—C18	116.1 (4)
C15—N4—C16	117.3 (4)	O2—C17—O1	124.8 (4)
C16—N4—Co1^iv^	121.3 (3)	O2—C17—C18	119.0 (4)
N1—C1—H1	118.0	C19—C18—C17	122.2 (4)
N1—C1—C2	123.9 (4)	C19—C18—C23	117.3 (4)
C2—C1—H1	118.0	C23—C18—C17	120.5 (4)
C1—C2—C6	119.8 (4)	C18—C19—H19	119.1
C3—C2—C1	116.6 (4)	C18—C19—C20	121.7 (4)
C3—C2—C6	123.6 (4)	C20—C19—H19	119.1
C2—C3—H3	119.9	C19—C20—H20	119.6
C4—C3—C2	120.3 (5)	C19—C20—C21	120.8 (4)
C4—C3—H3	119.9	C21—C20—H20	119.6
C3—C4—H4	120.5	C20—C21—C24	122.5 (4)
C5—C4—C3	119.0 (5)	C22—C21—C20	117.6 (4)
C5—C4—H4	120.5	C22—C21—C24	119.8 (4)
N1—C5—C4	122.5 (5)	C21—C22—H22	119.3
N1—C5—H5	118.8	C23—C22—C21	121.4 (4)
C4—C5—H5	118.8	C23—C22—H22	119.3
N2—C6—C2	110.4 (4)	C18—C23—H23	119.4
N2—C6—H6C	109.6	C22—C23—C18	121.1 (4)
N2—C6—H6D	109.6	C22—C23—H23	119.4
C2—C6—H6C	109.6	C21—C24—H24A	107.8
C2—C6—H6D	109.6	C21—C24—H24B	107.8
H6C—C6—H6D	108.1	H24A—C24—H24B	107.2
N2—C7—H7A	109.6	C25—C24—C21	117.8 (4)
N2—C7—H7B	109.6	C25—C24—H24A	107.8
N2—C7—C8	110.2 (4)	C25—C24—H24B	107.8
H7A—C7—H7B	108.1	C24—C25—H25A	108.4
C8—C7—H7A	109.6	C24—C25—H25B	108.4
C8—C7—H7B	109.6	C24—C25—C26	115.5 (4)
N3—C8—C7	111.5 (4)	H25A—C25—H25B	107.5
N3—C8—H8A	109.3	C26—C25—H25A	108.4
N3—C8—H8B	109.3	C26—C25—H25B	108.4
C7—C8—H8A	109.3	O3—C26—C25	117.4 (4)
C7—C8—H8B	109.3	O4—C26—O3	124.4 (4)
H8A—C8—H8B	108.0	O4—C26—C25	118.2 (4)
N3—C9—H9A	109.3		

Symmetry codes: (i) *x*−1/2, −*y*+1/2, *z*+1/2; (ii) −*x*+3/2, *y*−1/2, −*z*+3/2; (iii) *x*+1/2, −*y*+1/2, *z*−1/2; (iv) −*x*+3/2, *y*+1/2, −*z*+3/2.

### Poly[diaqua[µ2-1,4-bis(pyridin-3-ylmethyl)piperazine][µ2-4-(2-carboxylatoethyl)benzoato]cobalt(II)] Hydrogen-bond geometry (Å, º)

**Table:**

*D*—H···*A*	*D*—H	H···*A*	*D*···*A*	*D*—H···*A*
O5—H5*A*···N3^v^	0.89	2.20	2.980 (5)	146
O5—H5*B*···O4^i^	0.89	1.81	2.600 (5)	147
O6—H6*A*···O3^iv^	0.90	1.92	2.760 (4)	154
O6—H6*B*···O2	0.90	1.84	2.641 (4)	147

Symmetry codes: (i) *x*−1/2, −*y*+1/2, *z*+1/2; (iv) −*x*+3/2, *y*+1/2, −*z*+3/2; (v) −*x*+1/2, *y*−1/2, −*z*+3/2.
